# Involvement of Four Different Intracellular Sites in Chloroacetaldehyde- Induced Oxidative Stress Cytotoxicity

**Published:** 2012

**Authors:** Jalal Pourahmad, Mir-Jamal Hosseini, Mohammad Reza Eskandari, Faezeh Rahmani

**Affiliations:** a *Faculty of Pharmacy, Shaheed Beheshti University of Medical Sciences, Tehran.*; b *Department of Pharmacology and Toxicology, School of Pharmacy, Zanjan University of Medical Sciences, Zanjan, Iran.*

**Keywords:** Chloroacetaldehyde, Cytochrome P_450_, Hepatocyte, Mitochondrial/lysosomes cross-talk, Oxidative stress, Xanthine oxidase

## Abstract

Chloroacetaldehyde (CAA) is a chlorination by-product in finished drinking water and a toxic metabolite of a wide variety of industrial chemicals (*e.g*. vinyl chloride) and chemotherapeutic agents (*e.g*. cyclophosphamide and ifosfamide).

In this research, the cytotoxic mechanisms of CAA in freshly isolated rat hepatocytes were investigated.CAA cytotoxicity was associated with reactive oxygen species (ROS) formation and glutathione depletion suggesting that oxidative stress contributed to the CAA cytotoxic mechanism. CAA-induced oxidative stress cytotoxicity markers were significantly prevented by antioxidants, ROS scavengers, mitochondrial permeability transition (MPT) pore sealing agents, endocytosis inhibitors, ATP generators and xanthine oxidase inhibitor. In our study the hepatocyte mitochondrial membrane potential was rapidly decreased by CAA which was prevented by antioxidants and ROS scavenger indicating that mitochondrial membrane damage was a consequence of ROS formation. CAA cytotoxicity was also associated with lysosomal membrane rupture.

Our findings showed that at least four different intracellular sources including: metabolic enzymes cytochrome P_450_ and xanthine oxidase, mitochondrial respiratory chain disruption and lysosomal Haber-weiss reaction, were involved in CAA induced ROS formation and other subsequent cytotoxic events. Our other interesting finding was that the lysosomotropic agents prevented CAA induced mitochondrial membrane potential collapse and mitochondrial MPT pore sealing agents inhibited lysosomal membrane damage caused by CAA. It can therefore be suggested that there is probably a toxic interaction (cross-talk) between mitochondrial and lysosomal oxidative stress generating systems, which potentiates each organelle damage and ROS formation in CAA- induced hepatotoxicity.

## Introduction

With the advent of synthetic polymers in the industrial world, many chemicals are releasing into the environment in large quantities. Similarly, many chemicals are used as pesticides or as food preservatives have caused a series of environmental problems. Halogenated hydrocarbons are a main group of these environmental contaminants and some of these have been shown to be carcinogenic whereas others are mutagens and several of these compounds bind to DNA (1-2). Vinyl chloride, a major chemical, is used in the production of polyvinylchloride plastics and a correlation has been shown between workers exposed to vinyl chloride and development of angiosarcoma of the liver (3). 

It has been shown that vinyl chloride is metabolized in liver to a short-lived compound, chloroethylene oxide, which rapidly rearranges to CAA (3). Chloroacetaldehyde (C_2_H_3_Cl; CCA), an organic compound, is an alkylating agent and potent mutagen that specifically generates ethenobase DNA lesions (4-5).It is shown that CAA is major reactive metabolite of a vinyl chloride (6) and also ethylene dichloride (7) and ethylene chlorohydrins. CAA is a chlorination by-product in finished drinking water (8). The liver is one of the most important target tissues for CAA toxicity (9). CAA caused an increase in the absolute and relative organ weight, increase in liver tumors and preneoplastic lesions and also histologically mild hepatic effects (hepatic cytomegaly, necrosis, and chronic active inflammation) in mice (10). CAA induced a loss in viability of isolated rat hepatocytes in a concentration- and time-dependent manner (11). 

It already reported that cytosolic and mitochondrial rat liver aldehyde dehydrogenases play a significant role in CAA metabolism and also hepatocyte cytotoxicity was markedly enhanced when hepatocyte aldehyde dehydrogenase was inhibited prior to CAA addition (11-12). It was already reported that glutathione depletion, reversible thiol protein adduct formation (such as hemithioacetals or thioacetals), mitochondrial transmembrane potential collapse, fall of intracellular ATP levels and finally lipid peroxidation were involved in CAA-induced hepatocyte cytotoxicity (11, 13).Taken together, CCA decreased total glutathione and cellular ATP levels due to inhibition of oxidative phosphorylation at the level of complex I of the mitochondrial respiratory chain with large decrease in glucose synthesis from lactate due to inhibition of glyceraldehyde 3-phosphate dehydrogenase (14). It was also reported that hepatocytes from fasted rats were more susceptible to CAA toxicity. These *in- vitro *findings were also confirmed by *in-vivo *experiments (15).

However, all the intracellular pathways involved in CAA-induced oxidative stress hepatotoxicity have not yet been completely understood. In this study we investigated the cellular and molecular mechanisms involved in CAA-induced oxidative stress hepatotoxicity in isolated rat hepatocytes using accelerated cytotoxicity mechanisms screening (ACMS) techniques. 

In this investigation, we tested several classes of inhibitors and antagonists (based on our speculations and previous published works) including antioxidants (*α*- Tocopherol and butylated hydroxytoluene or BTH), radical scavengers (mannitol and DMSO), MPT pore sealing agents (carnitine and Trifluoperazine), endocytosis inhibitors (chloroquine and methylamine), ATP generators (fructose and L-glutamine), xanthine oxidase inhibitor (oxypurinol) as well as NADPH P_450_ reductase inhibitor (diphenyliodonium chloride) and reduced CYP2E1 inhibitor (phenylimidazole) for predicting of exact cellular and molecular mechanisms involved in CAA hepatotoxicity .

## Experimental

*Chemicals*

Chloroacetaldehyde (CAA), rhodamine 123, collagenase (from *Clostridium histolyticum*), bovine serum albumin (BSA), N-(2-hydroxyethyl)piperazine-N’-(2-ethanesulfonic acid) (HEPES), O-phthalaldehyde, Acridine orange, 2’,7’-dichlorofluorescin diacetate (DCFH-DA), Trichloroacetic acid, dimethyl sulfoxide (DMSO), 1-Bromoheptane, butylated hydroxytoluene, diphenyliodonium chloride, Trypan blue and heparin were purchased from Sigma-Aldrich Co. (Taufkrichen, Germany). All other chemicals were of the highest commercial grade available.

*Animals*

Male Sprague-Dawley rats weighing 280 to 300 g were housed in ventilated plastic cages over PWI 8-16 hardwood bedding. There were 12 air changes per hour, 12 h light photoperiod (lights on at 0800 h) and an environmental temperature of 21-23 °C with a 50-60% relative humidity. The animals were fed with a normal standard chow diet and tap water ad libitum. All experiments were conducted according to the ethical standards and protocols approved by the Committee of Animal Experimentation of Shaheed Beheshti University of Medical Sciences, Tehran, Iran.

*Isolation and incubation of hepatocytes*

Hepatocytes were obtained by collagenase perfusion of the liver and viability was assessed by plasma membrane disruption determined by trypan blue (0.2 w/v) exclusion test (16). Cells were suspended at a density of 10^6^ cells/mL in round-bottomed flasks rotating in a water bath maintained at 37°C in Krebs-Henseleit buffer (pH = 7.4), supplemented with 12.5 mM HEPES under an atmosphere of 10% O_2_, 85% N_2_, and 5% CO_2_ (17). Hepatocytes were pre-incubated for 30 min prior to addition of chemicals. Stock solutions of all chemicals (×100 concentrated for the water solutions or ×1000 concentrated for the methanolic solutions) were prepared fresh prior to use. To incubate CAA and all other water soluble treatments with the required concentration, we added 100 μL sample of its concentrated stock solution (×100 concentrated) to one rotating flask containing 10 mL hepatocyte suspension. For the chemicals, which dissolved in methanol, we prepared methanolic stock solutions (×1000 concentrated), and to achieve the required concentration in the hepatocytes, we added 10 μL samples of the stock solution to the 10 mL cell suspension. Ten microlitres of methanol did not affect the hepatocyte viability after 3 h incubation (data not shown). Glutathione (GSH) depleted hepatocytes were prepared by pre-incubation of hepatocytes with 200 μM 1-bromoheptane for 30 min as described by Khan and O’Brien, 1991 (18).

*Determination of EC*_50_ *for CAA*

To avoid either non-toxic or very toxic conditions in this study, we used EC_50_ concentrations for CAA. As described by Galati *et al*. 2000, the EC_50_ of a chemical in hepatocyte cytotoxicity assessment technique (with the total 3 h incubation period) is defined as the concentration, which decreases the hepatocyte viability down to 50% following the 2 h of incubation (19). All tests were performed three times. In order to determine EC_50_ 2h for CAA, dose-response curves were plotted and then EC_50 _was determined based on a regression plot of four different concentrations (50, 100, 200 and 400 μM) (data and curves not shown). Finally, the concentration of 300 μm (EC_50_) was selected for examination in our study.

*Cell viability*

The viability of isolated hepatocytes was assessed from the intactness of the plasma membrane as determined by the trypan blue (0.2% w/v) exclusion test (16, 20). Aliquots of the hepatocyte incubate were taken at different time points during the 3 h incubation period. 

*Determination of reactive oxygen species*

To determine the rate of hepatocyte reactive oxygen species (ROS) generation induced by CAA, dichlorofluorescin diacetate (DCFH-DA) was added to the hepatocytes. It penetrates hepatocyte cells and becomes hydrolyzed to non-fluorescent dichlorofluorescin (DCFH). The latter then reacts with ROS to form the highly fluorescent dichlorofluorescein (DCF), which effluxes the cell. The fluorescence intensity of DCF was measured using a Shimadzu RF5000U fluorescence spectrophotometer. Excitation and emission wavelengths were 500 and 520 nm, respectively. The results were expressed as fluorescent intensity per 10^6^ cells (21).

*Intracellular GSH and extra cellular GSSG assessment*

GSH and GSSG were determined according to the spectrofluorometric method (22). Each sample was meseared in quarts cuvettes using a fluorimeter set for 350 nm excitation and 420 nm emission wavelengths.

*Mitochondrial membrane potential assay*

Mitochondrial uptake of the cationic fluorescent dye, rhodamine123, has been used for estimation of mitochondrial membrane potential (23). The amount of rhodamine123 remaining in the incubation medium was measured fluorimeterically using a Shimadzu RF5000U fluorescence spectrophotometer set at 490 nm excitation and 520 nm emission wavelengths. The capacity of mitochondria to up take the rhodamine123 was calculated as the difference (between control and treated cells) in rhodamine123 fluorescence. Our data were shown as the percentage of mitochondrial membrane potential collapse (%ΔΨm) in all treated (test) hepatocyte groups (23).

*lysosomal membrane integrity assay*

Hepatocyte lysosomal membrane stability was determined from the redistribution of the fluorescent dye, acridine orange (17). Aliquots of the cell suspension (0.5 mL) that were previously stained with acridine orange (5 μM) were separated from the incubation medium by 1 min centrifugation at 1000 rpm. The cell pellet was then resuspended in 2 mL of fresh incubation medium. This washing process was carried out for two times to remove the fluorescent dye from the media. Acridine orange redistribution in the cell suspension was then measured fluorimetrically using a Shimadzu RF5000U fluorescence spectrophotometer set at 495 nm excitation and 530 nm emission wavelengths.

*Statistical analysis*

Levene’s test was used to check the homogeneity of variances. Data were analyzed using one-way analysis of variance (ANOVA) followed by Tukey’s HSD as the *post hoc *test. Results were presented as mean ± SD of triplicate samples. The minimal level of significance chosen was p < 0.05.

## Results

At least 80-90% of the control cells were viable following 3 h of incubation. The EC_50_2h concentration found for CAA was 300 μM. As shown in Table 1, CAA (300 μM) significantly increased hepatocyte membrane lysis comparing to control hepatocytes (p < 0.05). In addition to cytotoxicity ROS formation was significantly (p < 0.05) raised when hepatocytes were incubated with CAA at this EC50 2h concentration (Table 2). Both CAA induced cytotoxicity and ROS generation were prevented by antioxidants (*α*-Tocopherol and BHT), radical scavengers (mannitol and DMSO), MPT pore sealing agents (carnitine and Trifluoperazine), endocytosis inhibitors (chloroquine and methylamine), ATP generators (fructose and L-glutamine), xanthine oxidase inhibitor (oxypurinol) as well as by NADPH P_450_ reductase inhibitor (diphenyliodonium chloride) and reduced CYP2E1 inhibitor (phenylimidazole) (Tables 1, 2). 

**Table 1:** Effect of antioxidants, ROS scavengers, MPT pore sealing agents, lysosomotropic agents, ATP generators, xanthine oxidase inhibitor and CYP2E1 inhibitors on CAA induced hepatocyte lysis

**%Cytotoxicity**			**Addition**
**Incubation time**			
**3 h**	**2 h**	**1 h**	
22 ± 2	22 ± 2	18 ± 2	Control Hepatocytes
79 ± 5 ^a^	52 ± 4 ^a^	38 ± 3 ^a^	Chloroacetaldehyde (300 μM)
45 ± 4 ^b^	36 ± 3 ^b^	28 ± 3 ^b^	+*α*-Tocopherol succinat (10 μM)
43 ± 4 ^b^	36 ± 3 ^b^	27 ± 3 ^b^	+Butylatedhydroxytoluene (50 μM)
47 ± 4 ^b^	37 ± 3 ^b^	28 ± 3 ^b^	+Mannitol (50 mM)
48 ± 4 ^b^	38 ± 3 ^b^	29 ± 3 ^b^	+DMSO (150 μM)
45 ± 3 ^b^	35 ± 3 ^b^	26 ± 3 ^b^	+Carnitine (2 mM)
46 ± 4 ^b^	37 ± 3 ^b^	25 ± 3 ^b^	+Trifluoperazine (15 μM)
48 ± 3 ^b^	36 ± 3 ^b^	27 ± 3 ^b^	+Chloroquine (100 μM)
45 ± 4 ^b^	35 ± 3 ^b^	28 ± 3 ^b^	+Methylamine (30 mM)
48 ± 4 ^b^	38 ± 3 ^b^	28 ± 3 ^b^	+Fructose (10 mM)
47 ± 4 ^b^	38 ± 3 ^b^	29 ± 3 ^b^	+L-glutamine (1 mM)
46 ± 4 ^b^	36 ± 3 ^b^	29 ± 3 ^b^	+Oxypurinol (50 μM)
46 ± 3 ^b^	34 ± 3 ^b^	27 ± 4 ^b^	+Diphenyliodonium chloride (50 μM)
44 ± 4 ^b^	33 ± 3 ^b^	26 ± 3 ^b^	+Phenylimidazole (300 μM)
28 ± 3 ^b^	25 ± 2 ^b^	20 ± 3 ^b^	GSH depleted hepatocytes
92 ± 5 b	67 ± 5 ^b^	50 ± 4 ^b^	+ Chloroacetaldehyde (300 μM)

Hepatocytes (106 cells/mL) were incubated in Krebs–Henseleit buffer pH 7.4 at 37 °C for 3.0 h following the addition of CAA (300 μM). Cytotoxicity was determined as the percentage of cells that take up trypan blue (Moldeus *et al*. 1978). GSH depleted hepatocytes were prepared as described by Khan & O’Brien (1991). Values are expressed as mean ± SD of three separate experiments (n = 3) a:Significant difference in comparison with control hepatocytes (p < 0.05). b:Significant difference in comparison with CAA treated hepatocytes (p < 0.05)

**Table 2:** Effect of antioxidants, ROS scavengers, MPT pore sealing agents, lysosomotropic agents, ATP generators, xanthine oxidase inhibitor and CYP2E1 inhibitors on CAA induced ROS formation

**ROS formation (DCF)**						**Addition**
**Incubation time**						
**120 min**	**90 min**	**60 min**	**30 min**	**15 min**	**2 min**	
500 ± 16	480 ± 14	450 ± 14	440 ± 14	420 ± 14	410 ± 13	Control Hepatocytes
535 ± 16 ^a^	665 ± 18^a^	717 ± 19 ^a^	668 ± 18 ^a^	724 ± 19 ^a^	611 ± 18 ^a^	Chloroacetaldehyde (300 μM)
423 ± 14 ^b^	463 ± 14 ^b^	528 ± 16 ^b^	542 ± 16 ^b^	449 ± 14 b	440 ± 14 ^b^	+*α*-Tocopherol succinat (10 μM)
420 ± 14 ^b^	463 ± 15 ^b^	525 ± 14 ^b^	540 ± 16 ^b^	448 ± 15 ^b^	438 ± 14 ^b^	+Butylatedhydroxytoluene (50 μM)
440 ± 14 ^b^	447 ± 16 ^b^	550 ± 16 ^b^	426 ± 15 ^b^	428 ± 14 ^b^	375 ± 12 ^b^	+Mannitol (50 mM)
449 ± 14 ^b^	442 ± 14 ^b^	556 ± 16 ^b^	418 ± 14 ^b^	422 ± 14 ^b^	368 ± 11 ^b^	+DMSO (150 μM)
436 ± 14 ^b^	398 ± 11 ^b^	445 ± 16 ^b^	438 ± 16 ^b^	483 ± 14 ^b^	445 ± 14 ^b^	+Carnitine (2 mM)
438 ± 14 ^b^	449 ± 14 ^b^	446 ± 14 ^b^	439 ± 14 ^b^	468 ± 14 ^b^	421 ± 14 ^b^	+Trifluoperazine (15 μM)
438 ± 14 ^b^	449 ± 14 ^b^	427 ± 14 ^b^	428 ± 16 ^b^	461 ± 14 ^b^	490 ± 14 ^b^	+Chloroquine (100 μM)
445 ± 14 ^b^	469 ± 14 ^b^	402 ± 14 ^b^	430 ± 16 ^b^	434 ± 14 ^b^	494 ± 14 ^b^	+Methylamine (30 mM)
422 ± 14 ^b^	450 ± 14 ^b^	490 ± 18 ^b^	464 ± 14 ^b^	451 ± 14 ^b^	434 ± 14 ^b^	+Fructose (10 mM)
426 ± 16 ^b^	448 ± 15 ^b^	487 ± 16 ^b^	454 ± 16 ^b^	449 ± 15 ^b^	440 ± 14 ^b^	+L-glutamine (1 mM)
425 ± 14 ^b^	446 ± 14 ^b^	491 ± 18 ^b^	459 ± 14 ^b^	452 ± 15 ^b^	437 ± 14 ^b^	+Oxypurinol (50 μM)
430 ± 14 ^b^	450 ± 16 ^b^	520 ± 16 ^b^	476 ± 16 ^b^	411 ± 15 ^b^	432 ± 14 ^b^	+Diphenyliodonium chloride (50 μM)
436 ± 14 ^b^	449 ± 14 ^b^	527 ± 16 ^b^	479 ± 14 ^b^	403 ± 14 ^b^	411 ± 14 ^b^	+Phenylimidazole (300 μM)
513 ± 18 ^b^	488 ± 17 ^b^	468 ± 11 ^b^	449 ± 19 ^b^	426 ± 14 ^b^	412 ± 11 ^b^	GSH depleted hepatocytes
750 ± 19 ^b^	800 ± 18 ^b^	861 ± 19 ^b^	752 ± 19 ^b^	784 ± 19^b^	721 ± 19 ^b^	+Chloroacetaldehyde (300 μM)

Hepatocytes (10^6^ cells/mL) were incubated in Krebs–Henseleit buffer pH 7.4 at 37 °C for 3.0 h following the addition of CAA (300 μM). Dichlorofluorescein (DCF) formation was expressed as fluorescent intensity units (Pourahmad *et al*. 2010b). GSH depleted hepatocytes were prepared as described by Khan & O’Brien (1991). Values are expressed as mean ± SD of three separate experiments (n = 3).a:Significant difference in comparison with control hepatocytes (p < 0.05). b:Significant difference in comparison with CAA treated hepatocytes (p < 0.05).

Our results also showed that depleting hepatocyte glutathione (GSH) beforehand could increase the CAA induce hepatocyte cytotoxicity and ROS formation. All of the reagents used including antioxidants, radical scavengers, MPT pore sealing agents, endocytosis inhibitors, ATP generators, xanthine oxidase inhibitor, NADPH P_450_ reductase inhibitor, reduced CYP2E1 inhibitor and 1-bromoheptan (used for GSH depleting) did not significantly (p < 0.05) increase hepatocyte membrane lysis and ROS formation at concentrations used while incubated alone in isolated hepatocytes (Data not shown). 

As shown in Table 3, incubation of hepatocytes with CAA (300 μM) caused rapid hepatocyte GSH depletion. Most of the CAA induced hepatocyte GSH depletion could be attributed to the expulsion of GSSG (Table 3). Again pretreatment with antioxidants (*α*- Tocopherol and BTH), radical scavengers (mannitol and DMSO), MPT pore sealing agents (carnitine and Trifluoperazine), endocytosis inhibitors (chloroquine and methylamine), ATP generators (fructose and L-glutamine), xanthine oxidase inhibitor (oxypurinol), NADPH P_450_ reductase inhibitor (diphenyliodonium chloride) and reduced CYP2E1 inhibitor (phenylimidazole) significantly (p < 0.05) prevented both CAA induced hepatocyte intracellular GSH decrease and extracellular GSSG increase (Table 3). All of these protective agents did not show any significant effect (p < 0.05) on hepatocytes GSH/GSSG status at concentrations used (Data not shown).

**Table 3:** Effect of antioxidants, ROS scavengers, MPT pore sealing agents, lysosomotropic agents, ATP generators, xanthine oxidase inhibitor and CYP2E1 inhibitors on CAA induced glutathione depletion

**Extra cellular GSSG (μM) 3h**	**Intracellular GSH(μM) 3h**	**Addition**
11 ± 1	45 ± 4	Control Hepatocytes
22 ± 2 ^a^	21 ± 2 ^a^	+Chloroacetaldehyde (300 μM)
12 ± 1 ^b^	44 ± 3 ^b^	+α-Tocopherol succinat (10 μM)
13 ± 2 ^b^	43 ± 3 ^b^	+Butylatedhydroxytoluene (50 μM)
12 ± 1 ^b^	44 ± 3 ^b^	+Mannitol (50 mM)
13 ± 1 ^b^	44 ± 3 ^b^	+DMSO (150 μM)
12 ± 2 ^b^	42 ± 3 ^b^	+Carnitine (2 mM)
12 ± 2 ^b^	43 ± 3 ^b^	+Trifluoperazine (15 μM)
12 ± 1 ^b^	42 ± 2 ^b^	+Chloroquine (100 μM)
13 ± 2 ^b^	41 ± 2 ^b^	+Methylamine (30 mM)
12 ± 1 ^b^	43 ± 2 ^b^	+Fructose (10 mM)
11 ± 2 ^b^	42 ± 3 ^b^	+L-glutamine (1 mM)
12 ± 1 ^b^	41 ± 2 ^b^	+Oxypurinol (50 μM)
12 ± 1 ^b^	44 ± 3 ^b^	+Diphenyliodonium chloride (50 μM)
11 ± 1 ^b^	42 ± 3 ^b^	+Phenylimidazole (300 μM)

Hepatocytes (10^6^ cells/mL) were incubated in Krebs–Henseleit buffer pH 7.4 at 37 °C for 3.0 h following the addition of CAA (300 μM). Intracellular GSH and extra cellular GSSG were flurimetrically determined as described by Hissin & Hilf (1978). Values are expressed as mean ± SD of three separate experiments (n = 3).

a:Significant difference in comparison with control hepatocytes (p < 0.05). b:Significant difference in comparison with CAA treated hepatocytes (p < 0.05).

As shown in Table 4, CAA (300 μM) decreased mitochondrial membrane potential within 2 h of incubation which was prevented by antioxidants (*α*-Tocopherol and BTH) and radical scavengers (mannitol and DMSO) suggesting that CAA induced mitochondrial membrane potential decrease was subsequent of ROS formation. In addition xanthine oxidase inhibitor (oxypurinol), NADPH P450 reductase inhibitor (diphenyliodonium chloride) and reduced CYP2E1 inhibitor (phenylimidazole) inhibited decline of mitochondrial membrane potential. CAA induced hepatocyte mitochondrial membrane potential collapse was also potentiated when CAA was incubated in GSH depleted hepatocytes. All of these mentioned inhibitors did not show any significant effect (p < 0.05) on hepatocytes mitochondrial membrane potential at concentrations used while incubated alone (Data not shown). 

**Table 4:** Mitochondrial membrane potential decline during CAA induced hepatocyte injury

**%ΔΨm**						**Addition**
**Incubation time**						
**120 min**	**90 min**	**60 min**	**30 min**	**15 min**	**2 min**	
85 ± 5 ^a^	90 ± 5 ^a^	95 ± 5 ^a^	81 ± 4 ^a^	54 ± 3 ^a^	45 ± 4 ^a^	Chloroacetaldehyde (300 μM)
50 ± 4 ^b^	43 ± 3 ^b^	37 ± 3 ^b^	33 ± 3 ^b^	24 ± 3 ^b^	23 ± 3 ^b^	+α-Tocopherol succinat (10 μM)
49 ± 5 ^b^	43 ± 3 ^b^	38 ± 3 ^b^	32 ± 3 ^b^	24 ± 3 ^b^	24 ± 3 ^b^	+Butylatedhydroxytoluene (50 μM)
60 ± 5 ^b^	52 ± 4 ^b^	45 ± 3 ^b^	39 ± 3 ^b^	26 ± 3 ^b^	24 ± 3 ^b^	+Mannitol (50 mM)
58 ± 4 ^b^	50 ± 4 ^b^	45 ± 4 ^b^	38 ± 3 ^b^	27 ± 3 ^b^	25 ± 3 ^b^	+DMSO (150 μM)
62 ± 4 ^b^	55 ± 3 ^b^	49 ± 4 ^b^	42 ± 3 ^b^	32 ± 3 ^b^	29 ± 3 ^b^	+Carnitine (2 mM)
60 ± 4 ^b^	52 ± 3 ^b^	49 ± 4 ^b^	42 ± 3 ^b^	31 ± 2 ^b^	27 ± 3 ^b^	+Trifluoperazine (15 μM)
58 ± 4 ^b^	51 ± 4 ^b^	50 ± 3 ^b^	44 ± 3 ^b^	31 ± 3 ^b^	22 ± 3 ^b^	+Chloroquine (100 μM)
57 ± 4 ^b^	50 ± 4 ^b^	51 ± 4 ^b^	48 ± 3 ^b^	37 ± 3 ^b^	21 ± 3 ^b^	+Methylamine (30 mM)
51 ± 4 ^b^	44 ± 4 ^b^	38 ± 3 ^b^	31 ± 3 ^b^	30 ± 3 ^b^	24 ± 3 ^b^	+Fructose (10 mM)
50 ± 4 ^b^	42 ± 3 ^b^	35 ± 3 ^b^	30 ± 3 ^b^	27 ± 3 ^b^	26 ± 3 ^b^	+L-glutamine (1 mM)
48 ± 3 ^b^	40 ± 4 ^b^	33 ± 4 ^b^	28 ± 3 ^b^	27 ± 3 ^b^	25 ± 3 ^b^	+Oxypurinol (50 μM)
47 ± 3 ^b^	46 ± 3 ^b^	44 ± 3 ^b^	42 ± 3 ^b^	31 ± 3 ^b^	22 ± 3 ^b^	+Diphenyliodonium chloride (50 μM)
48 ± 4 ^b^	45 ± 4 ^b^	41 ± 4 ^b^	39 ± 3 ^b^	31 ± 3 ^b^	20 ± 3 ^b^	+Phenylimidazole (300 μM)
11 ± 5 ^b^	9 ± 4 ^b^	8 ± 3 ^b^	7 ± 2 ^b^	6 ± 2 ^b^	5 ± 1 ^b^	GSH depleted hepatocytes
100 ± 4 ^b^	98 ± 5	96 ± 5	94 ± 5 ^b^	66 ± 5 ^b^	71 ± 5 ^b^	+ Chloroacetaldehyde (300 μM)

Hepatocytes (10^6^ cells/mL) were incubated in Krebs–Henseleit buffer pH 7.4 at 37 °C for 3.0 h following the addition of CAA (300 μM). Mitochondrial membrane potential was determined as the difference in mitochondrial uptake of the rhodamine 123 between control (immediately after preparation *i.e*. at time zero min) and treated cells at time sampled. Our data were shown as the percentage of mitochondrial membrane potential collapse (%ΔΨm) in all treated (test) hepatocyte groups (Andersson *et al*.1987). GSH depleted hepatocytes were prepared as described by Khan & O’Brien (1991). Values are expressed as mean ± SD of three separate experiments (n = 3). a: Significant difference in comparison with control hepatocytes (p < 0.05). b: Significant difference in comparison with CAA treated hepatocytes (p < 0.05).

When hepatocyte lysosomes were loaded with acridine orange (a lysosomotropic agent), a significant release of acridine orange into the cytosolic fraction ensued within 120 min of incubation with CAA indicating a severe damage to lysosomal membrane (Table 5). CAA- induced acridine orange release was again prevented by antioxidants (*α*-Tocopherol and BTH), radical scavengers (mannitol and DMSO), ATP generators (fructose and L-glutamine), xanthine oxidase inhibitor (oxypurinol), NADPH P_450_ reductase inhibitor (diphenyliodonium chloride) and reduced CYP2E1 inhibitor (phenylimidazole) (Table 5). Again depleting hepatocyte GSH beforehand potentiated the acridine orange loaded hepatocytes against CAA- induced acridine orange release. All of these inhibitors did not show any significant effect (p < 0.05) on acridine orange redistribution from lysosomes to cytosol at concentrations used when incubated alone in isolated hepatocytes (Data not shown).

**Table 5:** Lysosomal membrane integrity changes during CAA induced hepatocyte injury

**% Acridine orange redistribution**						**Addition**
**Incubation time**						
**120 min**	**90 min**	**60 min**	**30 min**	**15 min**	**2 min**	
40 ± 4 ^a^	44 ± 4 ^a^	37 ± 3 ^a^	45 ± 4 ^a^	44 ± 4 ^a^	40 ± 4 ^a^	Chloroacetaldehyde (300 μM)
25 ± 2 ^b^	25 ± 2 ^b^	23 ± 2 ^b^	25 ± 2 ^b^	27 ± 3 ^b^	21 ± 2 ^b^	+α-Tocopherol succinat (10 μM)
25 ± 2 ^b^	24 ± 2 ^b^	22 ± 2 ^b^	25 ± 1 ^b^	26 ± 3 ^b^	22 ± 2 ^b^	+Butylatedhydroxytoluene (50 μM)
23 ± 2 ^b^	22 ± 1 ^b^	23 ± 2 ^b^	28 ± 3 ^b^	26 ± 3 ^b^	25 ± 3 ^b^	+Mannitol (50 mM)
23 ± 3 ^b^	23 ± 2 ^b^	25 ± 2 ^b^	28 ± 3 ^b^	27 ± 3 ^b^	26 ± 3 ^b^	+DMSO (150 μM)
28 ± 2 ^b^	30 ± 2 ^b^	27 ± 2 ^b^	29 ± 3 ^b^	28 ± 3 ^b^	25 ± 2 ^b^	+Carnitine (2 mM)
20 ± 2 ^b^	21 ± 2 ^b^	20 ± 2 ^b^	25 ± 2 ^b^	27 ± 3 ^b^	21 ± 2 ^b^	+Trifluoperazine (15 μM)
25 ± 2 ^b^	25 ± 2 ^b^	28 ± 3 ^b^	28 ± 2 ^b^	22 ± 2 ^b^	24 ± 2 ^b^	+Chloroquine (100 μM)
29 ± 3 ^b^	28 ± 3 ^b^	25 ± 2 ^b^	28 ± 3 ^b^	22 ± 2 ^b^	23 ± 2 ^b^	+Methylamine (30 mM)
23 ± 2 ^b^	22 ± 2 ^b^	26 ± 3 ^b^	25 ± 2 ^b^	27 ± 3 ^b^	27 ± 2 ^b^	+Fructose (10 mM)
21 ± 2 ^b^	24 ± 2 ^b^	25 ± 2 ^b^	23 ± 2 ^b^	17 ± 2 ^b^	16 ± 2 ^b^	+L-glutamine (1 mM)
28 ± 2 ^b^	28 ± 3 ^b^	27 ± 4 ^b^	27 ± 2 ^b^	26 ± 2 ^b^	25 ± 2 ^b^	+Oxypurinol (50 μM)
30 ± 3 ^b^	26 ± 3 ^b^	28 ± 3 ^b^	26 ± 2 ^b^	22 ± 2 ^b^	21 ± 2 ^b^	+Diphenyliodonium chloride (50 μM)
28 ± 3 ^b^	25 ± 2 ^b^	28 ± 3 ^b^	25 ± 2 ^b^	20 ± 2 ^b^	21 ± 2 ^b^	+Phenylimidazole (300 μM)
11 ± 2 ^b^	9 ± 2 ^b^	8 ± 2 ^b^	6 ± 2 ^b^	4 ± 1 ^b^	3 ± 2 ^b^	GSH depleted hepatocytes
76 ± 5 ^b^	74 ± 5	68 ± 5	58 ± 4 ^b^	56 ± 4 ^b^	51 ± 4 ^b^	+ Chloroacetaldehyde (300 μM)

Hepatocytes (10^6^cells/mL) were incubated in Krebs–Henseleit buffer pH 7.4 at 37 °C for 3.0 h following the addition of CAA (300 μM). Lysosomal membrane damage was determined as difference in redistribution of acridine orange from lysosomes into cytosol between control cells (immediately after preparation *i.e. *at time zero min) and treated cells at time sampled. Our data were shown as the percentage of lysosomal membrane leakiness in all treated (test) hepatocyte groups (Pourahmad *et al. *2010a). GSH depleted hepatocytes were prepared as described by Khan & O’Brien (1991). Values are expressed as mean ± SD of three separate experiments (n = 3). a: Significant difference in comparison with control hepatocytes (p < 0.05). b: Significant difference in comparison with CAA treated hepatocytes (p < 0.05).

The interesting finding was that CAA induced hepatocyte mitochondrial membrane potential collapse prevented by lysosomotropic agent (chloroquine and methylamine) and on the other hand lysosomal membrane leakiness induced by CAA were also prevented by mitochondrial MPT pore sealing agents (carnitine and Trifluoperazine) (Tables 4 and 5).

## Discussion

Chloroacetaldehyde (CAA) is a toxic metabolite of a wide variety of industrial chemicals (*e.g*. vinyl chloride) and chemotherapeutic agents (*e.g. *cyclophosphamide and ifosfamide) (24-25).In previous studies, this agent showed toxic effects in liver, kidney and neurons (14, 26). However, the precise molecular and cellular mechanism of CAA cytotoxicity is still unclear. Based on the previous published works, CAA acts as an alkylating agent and could produce inter-strand cross-links and inhibit DNA-synthesis (4). It was already reported that the molecular mechanism of CAA-induced cytotoxicity is associated with rapid depletion of protein thiols, inhibition of mitochondrial respiration with ATP-depletion and lipid peroxidation leading to cell death (11). It was also shown that CAA toxicity was mainly due to cellular glutathione, disturbed Ca^2+^ signaling, caspase-3 activation, reduced cellular protein content and finally disruption of cell membrane integrity. (13, 26-27).

These effects are closely related with the disturbance of cellular energy metabolism and depletion of cellular thiol compounds, *i.e*. glutathione and coenzyme A derivatives that ultimately result in cellular oxidative damage (11, 28). Our result showed that ROS scavengers (DMSO) and antioxidants (BTH) significantly protected the hepatocytes against CCA-induced GSH depletion and other oxidative stress cytotoxicity markers.

It is proven that oxidative stress establish with an imbalance between the production and removal of ROS. Therefore, overproduction of these substances or significant depletion of antioxidant defense molecules (*e.g*. GSH) causes oxidative stress in different cells (29). It is well known that complex I is susceptible for free radical release and CAA, a reactive metabolite produced both in the liver and kidney of ifosfamide -treated patients, may inhibit complex I activity and stimulate oxygen consumption, fatty acid oxidation and reactive oxygen species formation (30). Our results also showed that CAA induces hepatocyte mitochondrial membrane potential collapse and ROS formation. Besides, when isolated hepatocytes were incubated with CAA, glutathione depletion was occurred as a consequence of ROS formation. Therefore, it can be concluded that the cytotoxic mechanism of CAA is mediated by oxidative stress. Our study also shows that hepatocytes could be protected by the pretreatment of several free radical scavengers (Mannitol, DMSO) and antioxidants (*α*-Tocopherol, BHT) against CCA-induced cytotoxicity.

It has already been reported that acetaldehyde-induced lipid peroxidation is attributed to oxygen activation when xanthine oxidase utilizes acetaldehyde as a substrate (31-32). Furthermore oxypurinol, an allopurinol metabolite and xanthine oxidase inhibitor, protected against hepatocyte CAA toxicity, suggesting that CAA is a substrate for xanthine oxidase and that superoxide radicals formed by its metabolizing enzyme, xanthine oxidase may be involved in CAA-induced lipid peroxidation. Another suggestion is that hepatocytes oxidize aldehydes to aldehyde peroxy radicals, peracids or hydroxyl radicals, which initiate extensive oxidative stress and lipid peroxidation (33). 

On the other hand, CYP2E1 is one the most powerful inducers of oxidative stress in liver cells (34). CYP2E1 itself is also an effective enzyme for ROS production, exhibiting enhanced NADPH oxidase activity, and elevated rates of production of O_2_**.**^─^ and H_2_O_2_ even in the absence of substrate (35-37). In our study CAA- induced ROS formation was protected by four different intracellular pathways inhibitors including: inhibitors of CYP2E1 (phenylimidazole) and NADPH P_450_ reductase (diphenyliodonium chloride), xanthine oxidase inhibitor (oxypurinol), mitochondrial pore sealing agents (carnitine and Trifluoperazine) and lysosomotropic agents (chloroquine and methylamine) suggesting that at least four different intracellular sources: cytochrome P_450_, xanthine oxidase, metal destructive interaction, mitochondrial electron transfer chain disruption and lysosomal Haber–Weiss reaction are involved in CAA induced ROS formation and its subsequent cytotoxic events. 

Our results showed that hepatocyte mitochondrial membrane potential was rapidly decreased by CAA .CAA induced mitochondrial membrane decrease was prevented by ROS scavengers, antioxidants, mitochondrial ATP generator (glutamine), MPT pore sealing agents (carnitine) and glycolytic substrates (fructose) indicating that mitochondrial membrane damage was a consequence of ROS formation and cellular energy depletion. Furthermore, the ATP generators prevented CAA- induced cytotoxicity and ROS generation suggesting that the collapse may be a consequence of MPT pore opening and ATP depletion. The ΔΨm is maintained by continuous pumping of protons from the matrix across the inner mitochondrial membrane into the inter-membrane space. Because these protons in turn are used to drive the ATP synthase, a collapse of the ΔΨm invariably results in compromised ATP synthesis (38). Any damage to mitochondrial ATP generation results in intracellular acidosis and osmotic injury. The later is the cause of plasma membrane lysis (39).

**Figure 1:**
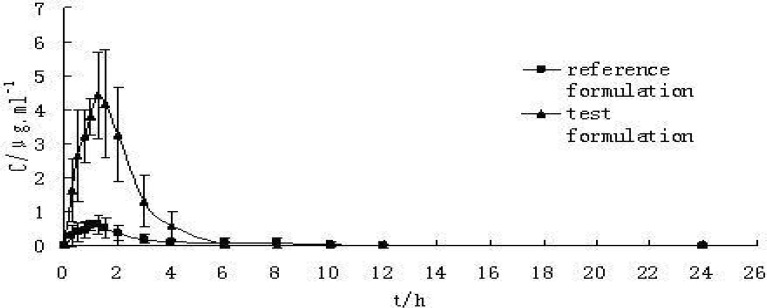
Proposed mechanism for chloroacetaldehyde (CAA) induced hepatocyte toxicity**- **Bid and Bax- two pro-apoptotic proteins, MPT pore- mitochondrial permeability transition pore, PLA_2_- phospholipase A_2_, ROS- reactive oxygen species, SOD2- mitochondrial superoxide dismutase-2

Lysosomes are one of the intracellular sources for ROS generation in toxic conditions (40). Haber-weiss reaction occurs in the lysosomes and catalyzed by intralysosomal redox-active iron that leads to ROS formation. In our study CAA caused lysosomal membrane damage that was again prevented by antioxidants and radical scavengers suggesting that lysosoms are one of the ROS production sites in CAA toxicity. Our other interesting results were that the lysosomotropic agents prevented CAA-induced mitochondrial membrane potential collapse and mitochondrial MPT pore sealing agents inhibited lysosomal membrane damage caused by CAA. It can therefore be suggested that there is probably a toxic interaction between mitochondrial and lysosomal oxidative stress generating systems, which potentiates each organelle damage and ROS formation in CAA model of hepatotoxicity (Figure 1).

Metabolic activation of CAA through cytochrome P_450 _monooxygenase (CYP2E1) and xanthine oxidase leads to considerable free radical formation. Increased ROS formation could directly damage hepatocyte mitochondria via MPT pore opening, cytochrome c expulsion and disruption of electron transfer chain. Hydrogen peroxide (H_2_O_2_) originated either from CAA metabolic activation or damaged mitochondria diffuses into lysosomes (due to lipophilic nature) and a Fenton type reaction (Haber- weiss) catalyzed by intra-lysosomal redox-active Fe^2+^/Fe^3+^ occurs. This leads to highly reactive hydroxyl radical (HO^.^) generation. Hydroxyl radicals could destabilize the lysosomal membrane integrity and release of digestive proteases (*i.e*. cathepsins). These released proteases and hydroxyl radicals could either open the mitochondrial MPT pore via oxidation of surrounding thiol groups or through activation of Bid or Bax pro-apoptotic proteins and other lytic enzymes including phospholipase A_2_ (PLA_2_). Disruption of electron transfer chain potentiates mitochondrial H_2_O_2_ generation and continues the cycle of mitochondrial/lysosomal oxidative stress toxic cross-talk that accelerates CAA hepatocyte toxicity (Figure 1).

## Conclusions

In our study CYP2E1 inhibitor and xanthine oxidase inhibitor perfectly prevented toxicity markers including: cytotoxicity, ROS formation, GSH depletion, mitochondrial membrane potential decrease and lysosomal membrane rupture. Therefore, it can be suggested that shared metabolism of CAA with CYP2E1 and xanthine oxidase is a primary event in CAA toxicity that leads to ROS formation. Excessive ROS targets mitochondria and lysosomes and causes oxidative stress membrane damages in both sub-organelles. Finally, mitochondrial/ lysosomal toxic cross-talk potentiates oxidative stress that accelerates CAA hepatocyte cytotoxicity.
